# Sepsis in patients with severe TBI: a retrospective CT scoring study

**DOI:** 10.1186/s12245-025-00911-6

**Published:** 2025-06-23

**Authors:** Guang-Sheng Wang, Da-Zhi Zhou, Shao-Dan Wang, Ye-Ting Zhou, Dao-Ming Tong

**Affiliations:** 1 Department of Neurology, Affiliated Shuyang Hospital of Xuzhou Medical University, Jiangsu, China; 2 Department of Neurosurgery, Affiliated Shuyang Hospital of Xuzhou Medical University, Jiangsu, China; 3 Department of Intensive Care Medicine, Affiliated Shuyang Hospital of Xuzhou Medical University, Jiangsu, China; 4 Department of General Surgery, Affiliated Shuyang Hospital of Xuzhou Medical University, Jiangsu, China; 5 Medical Research Department, Affiliated Shuyang Hospital of Xuzhou Medical University, No. 9, Yingbin Road, Shu town, Jiangsu, 223600 China

## Abstract

**Background:**

Multiple organ failure (MOF) is a severe complication associated with high mortality in sepsis after severe TBI (sTBI).

**Objective:**

To investigate the usefulness of a rapid computed tomography (CT) screening score for predicting of mortality and outcomes of sepsis after sTBI.

**Methods:**

We retrospectively analyzed those data of patients who were admitted to the ICU. All sTBI patients with or without sepsis underwent rapid CT screening before ICU admission and were admitted to the ICU for > 24 h were included in this study. The main outcome was sepsis-related the mortality after sTBI. The secondary outcome was the GOSE score during the first 60 days.

**Results:**

Among a random sample of 412 adult patients with sTBI, we found 249 sepsis after sTBI (60.4%) and 163 (39.6%) non- sepsis after sTBI events. The main organ failure was early brain (94.8%) and lung injury(91.2%) caused by community-acquired pnumonia (CPA). The CT score was higher in the sTBI with sepsis group than in the sTBI without sepsis group(wean 3.5 score vs. 0.9 score, *p* < 0.001).The SOFA score was also higher in the sTBI with sepsis group than in the sTBI without sepsis group(wean 5.9 score vs. 3.6 score, *p* < 0.001). The risk of death for sepsis after sTBI was an elevated CT score (hazard ratio[HR], 4.6; 95% confidence interval[CI], 3.373–10.49; *p* < 0.001) and an elevated SOFA score (HR,3.0; 95% CI, 2.054–4.826; *p* < 0.001).The area under the ROC curve for mortality was significantly larger for the elevated CT score (0.90, 95%CI 0.86–0.97 ) than for the elevated score (0.85, 95%CI 0.81–0.89 ) (*P* < 0.001). The elevated CT score in the area under the ROC curve for mortality was with 97.0% of sensitivity and 100.0% of specificity. At 60 days follow-up, the risk of death for sepsis after sTBI was higher than those non- sepsis after sTBI (*p* < 0.001).

**Conclusions:**

Elevated CT score is a well indicator of high incidence and mortality for sepsis after sTBI in the ICU, which suggests that this very current and practical event is involved to a global health care problem. But it could still need further verification in future investigation.

**Supplementary Information:**

The online version contains supplementary material available at 10.1186/s12245-025-00911-6.

## Introduction

In the United States, acute traumatic brain injury (TBI) is a major health care problem [1]. Also, the incidence rate of TBI patients with high infection risk has increased [2, 3], so comprehensive treatment strategies are needed to treat infection. In particular, the number of patients who had a sepsis after severe TBI has been increasing [3], and the mortality rate of sepsis was very high [4], which indicates that the high mortality rate of sepsis after severe TBI (sTBI) may be a complex problem. However, the main risk factors affecting the mortality rate of sepsis after severe TBI, especially CT screening score and sepsis mortality, was still unclear. The previous consensus definition of sepsis is at least one organ failure and signs that meet two or more criteria of systemic inflammatory response syndrome (SIRS) [5]. The third international consensus definition working group redefined sepsis is a life-threatening organ dysfunction caused by host dysregulation of the response to infection [6]. Therefore, SIRS is no longer considered as a diagnostic criterion for possible sepsis in ICU. For a long time, the Sequential Organ Failure Assessment (SOFA) standard has been used to screen for sepsis related six types of organ dysfunction [7]. However, the SOFA standards can only be used on ICU admission. In this study, we hypothesized that sepsis after sTBI may have a high prevalence of life-threatening multiple organ injury, and at least 4 to 5 of the acute organ injuries and site of infection are more likely to be determined by a rapid brain + chest + abdominal CT screening before ICU admission. The purpose of this study is to determine whether the incidence and mortality of sepsis after sTBI is involved elevated CT screening score. We hope that our study results will verity this hypothesis and provide evidence of predicting poor outcome for sepsis after sTBI in the ICU, so that to improve early diagnosis and management.

## Methods

### Study design and population

We retrospectively studied the data of adult sTBI patients (initial GCS score ≤ 8) admitted to general ICUs in a tertiary teaching hospital. This hospital is a specialty hospital with a referral centre (equipped with 10 ambulances) responsible for all critical or emergency work in northern counties of China. Therefore, almost all sepsis after sTBI patients were directly sent to the intensive care unit(ICU), which operates 24 h a day. We collected available data of sepsis after sTBI patients who underwent prospective primary diagnosis registration in the ICU from January 2016 to December 2019 having a post hoc likelihood of infection rated at least probable, according to definitions published previously. Sepsis was defined as a clinically suspected infection with a sequential organ failure assessment score of 2 or greater at ICU admission, approximating the sepsis-3 definitions. Patients in whom the onset of infection had occurred more than 2 days before ICU admission were not eligible for inclusion. We excluded those patients had any potential infection symptoms before the trauma and those patients did not underwent brain + chest + abdominal CT screening before ICU admission. We also excluded moderate TBI (GCS score > 9) and those TBI patients who died within the first 3 h or were removed from the ICU due to without detailed record of information. The research scheme was approved by the Institutional review board of Shuyang Hospital affiliated to Xuzhou Medical University and was carried out in accordance with the Declaration of Helsinki of 1964 (revised in 2008). Due to the retrospective nature of this study, the necessity of informed consent was waived (ID: 2016-N-0001).

### Data collection and related criteria

According to the data in the hospital registration database, this study included adult patients with sTBI with an initial GCS score of ≤ 8, and underwent brain + chest + abdominal CT scans before ICU admission. We collected reports and descriptions from two radiologists with double blind reading(individually) which was previously diagnosed before in the ICU. A senior neurologist reviewed and registered these CT reports. The diagnosis of sepsis was based on the sepsis-3 criteria. Therefore, for the diagnosis of sepsis, we mainly focus on identifying the infection site and at least one of the six organ injury due to infection using early systemic CT screening before ICU admission.

According to our brain CT screening before ICU admission, the features of CT images related brain injury [8–10], which indicates ischemic/hemorrhagic injury [8] with or without acute brain edema and brain hernia [9, 10]. We can like to be classified as CT scores of 1–2: 1 score refer to ischemic/hemorrhagic/inflammatory injury with or without acute brain edema, but no brain hernia, 2 scores refer to ischemic/hemorrhagic/inflammatory injury with brain hernia. According to current CT features of acute respiratory distress syndrome (ARDS) [11], the diagnostic criteria of ARDS is classified as bilateral opacities for imaging criteria. In this study, we used a quantifying CT score for diagnosis of ARDS as having scores of 1–2: 1 score indicated as unilateral opacities, 2 scores indicated as bilateral opacities/ARDS. In addition, the CT score of intra-abdominal sepsis can also be classified as CT scores of 1–2: 1 score as without peritonitis [12], and 2 score as with peritonitis.

Delays in diagnosis and treatment could lead to increased mortality [6]. Based on rapid systemic CT screening before ICU admission, early diagnosis of sepsis, including primary and secondly complications, must have one to two of the brain + lung + abdominal acute organ injury due to infection. In this study, we described and defined a diagnosis criteria for rapid systemic CT scoring with or without sepsis as follow: the total score of brain + lung + abdominal CT scoring as 6 scores. A low CT score indicate of 1–2 without sepsis, and a high CT score as of 3–6 with sepsis.

The infection of before ICU admission mainly indicated CAP. CAP is characterized by airspace merging of one segment or lobe, especially the lower lobe, rather than pulmonary contusion or other non-infectious causes. However, severe CAP usually rapidly develops into ARDS, which is manifested as opacities like shadows that affect bilateral lungs. The manifestation of pneumonia was confirmed by chest CT screening before ICU admission.

In this population, most patients initially experience sudden coma due to acute TBI (GCS score < 8 = SOFA score ≥ 2). Therefore, we also used sepsis related SOFA scores to evaluate sepsis related organ dysfunction. SOFA score ≥ 2 is defined as acute organ failure for specific organs. Previous studies have shown that SIRS mainly involves the release of a large number of inflammatory cytokines. The SIRS ≥ 2 points indicate potential infection.

### Outcomes assessment

The main outcome was the mortality of sepsis after sTBI. The secondary outcome was the functional outcome determined by using the Glasgow Outcome Scale Extension (GOSE) [12]. The GOSE is divided into two categories: the favourable (score 4–8) and unfavourable (score 1–3) outcomes (Table S2 in the Supplement). To investigate the results of the patient’s first 60 days of hospitalization, survival results were initially determined from hospital records. If the hospital stay was less than 60 days and the patient dies after discharge, GOSE information was obtained through telephone interviews with the patient or their closest living relatives, and follow-up was conducted 60 days after the injury. The follow-up started in December 2019 and ended in January 2020.

### Statistical analyses

The results of each group are expressed as the mean ± standard deviation (SD) or median (IQR), with n (%) representing qualitative values. Continuous variables were compared using the t test. If multivariable adjusted hazard ratio (HR) and 95% confidence interval (CI) were significant in univariate analysis, a Cox proportional risk model was used to check the baseline status of sepsis and determine whether these variables play a role in the risk of death events. The Kaplan‒Meier method was used to construct a cumulative survival event curve for outcomes. The initial 60 day crude event occurrence rate was determined by the Kaplan‒Meier ratio. The functional outcome determined by using the Glasgow Outcome Scale Extension (GOSE) [12]. In addition, comparisons of the areas under the receiver operating characteristic (ROC) curves were also performed. If the bilateral *p* value was less than 0.05, the difference between patients was considered significant. Proprietary computerized statistical software package (SPSS 17.0.) was used for statistical calculations.

## Results

**Fig. 1:**
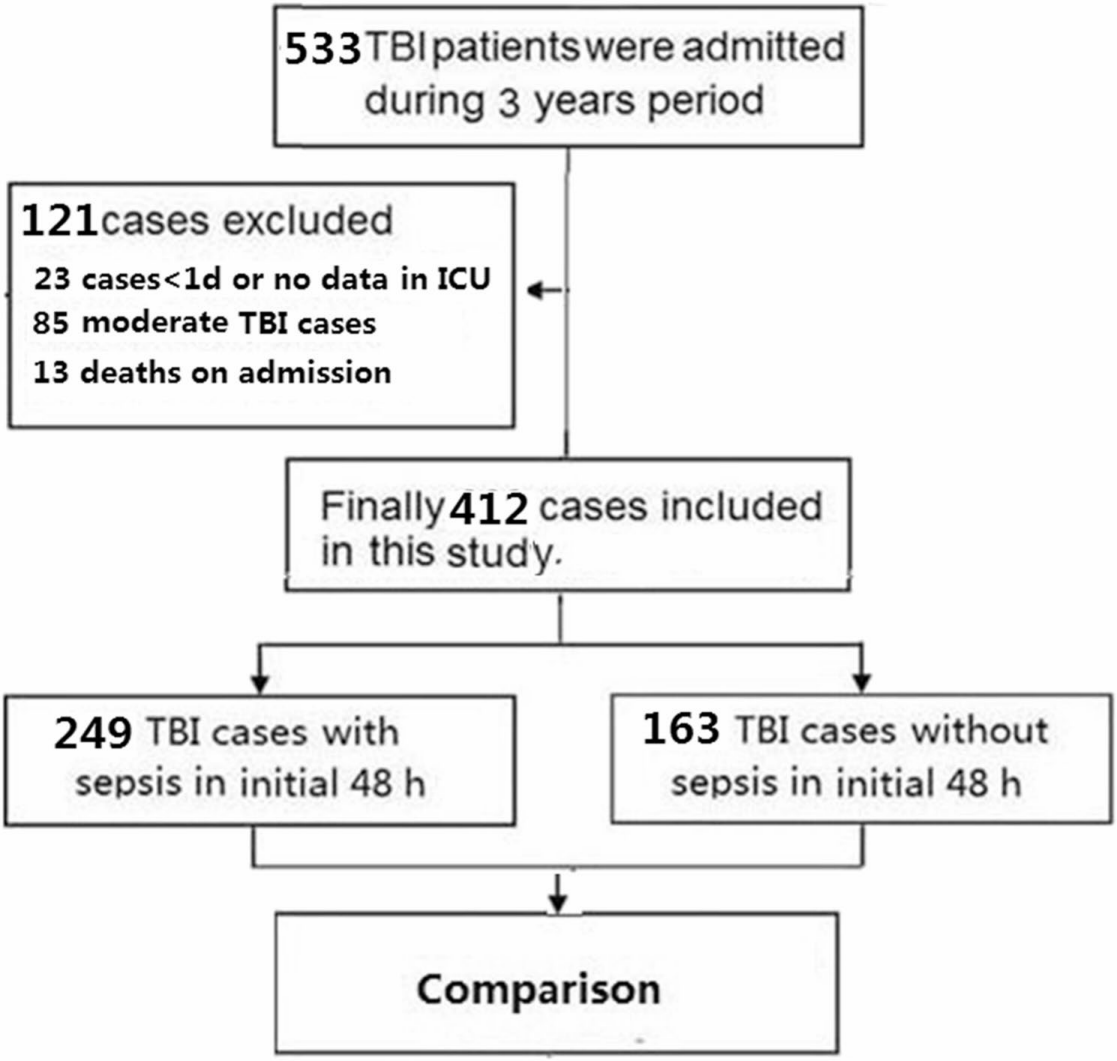
Flow chart. The patients who were not met the eligibility for this study design were excluded. Finally, 412 TBI in ICU admissions were included in the present study

During the study period, a total of 533 sTBI patients within random sample were admitted to our ICU. According to our screening criteria, 121 patients were excluded, including TBI patients (*N* = 23) who were hospitalized in the intensive care unit for less than 1 day or had no medical data, moderate TBI patients (*N* = 85), and sTBI patients who were considered dead at admission through prehospital Cardiopulmonary resuscitation (*N* = 13). Finally, 412 sepsis after sTBI were included in this study (Fig. 1).

**Fig. 2:**
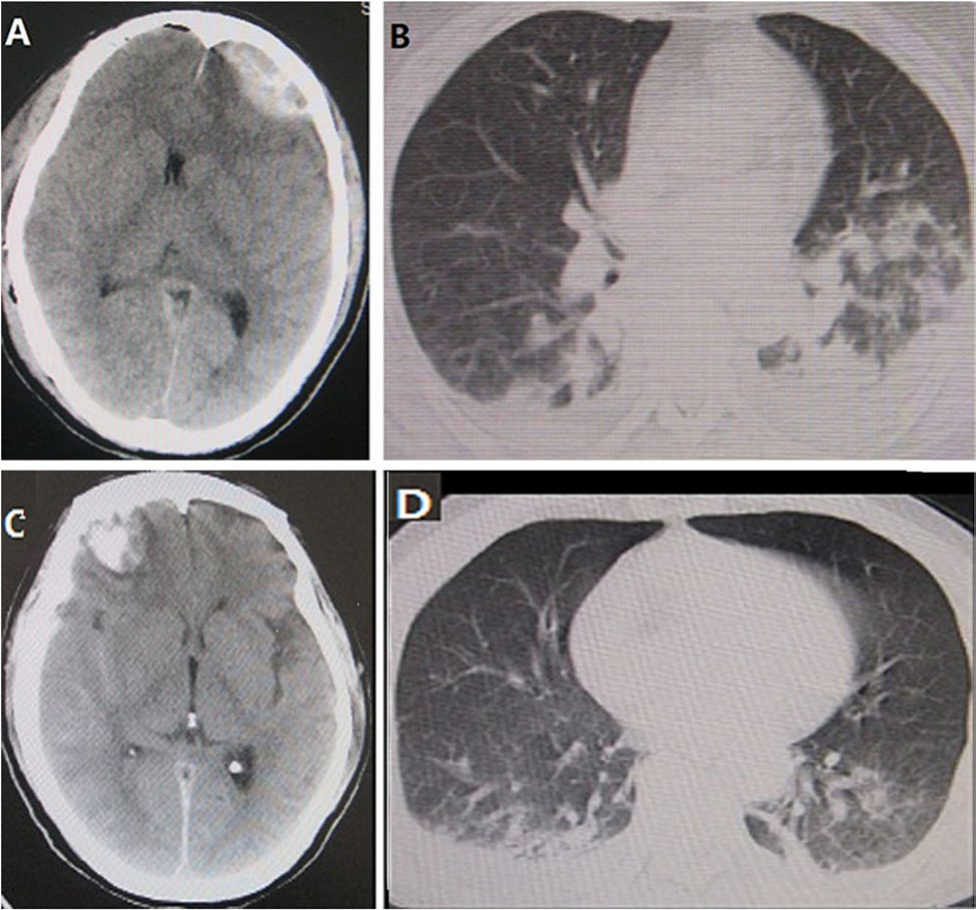
CT images of pneumonia caused brain edema following TBI. Fig **A** and **B** represent an adult patient with diffuse or vasogenic brain edema due to pneumonia with later positive sputum culture following TBI. Fig **C** and **D** represent an adult patient with diffuse brain edema caused by pneumonia with later positive sputum culture following TBI

Baseline characteristics of sTBI is showed in sTable 3 in the Supplement. The median age of TBI patients was 53 years old; there were 315 males (76.4%). The median time to directly arrive at the ICU after injury was 1 h. Road traffic collisions accounted for 59.9% of the TBI cases, falls accounted for 35.4%, and injuries with unknown causes accounted for 4.6%. The characteristics of sepsis after sTBI patients is shown in Table 1. Among 249 sepsis after sTBI patients, the percent of sepsis related acute brain injury was 94.8% (236/249), and the percent of sepsis related ALI was 91.2% (227/249). Before on ICU, rapid CT screening showed that the majority of sTBI patients (89.2%, 222/249) had evidence of acute brain and lung injury caused by pneumonia (see Fig. 2). Most patients with sTBI had early infection events before ICU admission. The most common cause of infection was CAP (79.1%, 197/249), followed by skin laceration/wound infection (20.9%). The early onset of most CAP (median time: 1 h before ICU admission) was related to sepsis which was confirmed by CT (Fig. 2). The univariate analysis results of sepsis after sTBI and non- sepsis after sTBI are shown in Table 2. Among 412 patients, 249 (60.4%) had sepsis events, and 163 (39.6%) had not sepsis events. There were no significant differences in age, gender, and time from trauma to intensive care unit between the two groups. We found that there was significantly difference in GCS score (5.0 ± 1.8 vs. 6.0 ± 1.9, *p* = 0.027), SOFA score (5.9 ± 2.2 vs. 3.6.0 ± 0.5, *p* < 0.001), Elevated CT score (3.5 ± 1.4 vs. 0.9 ± 1.1., *p* < 0.001), SIRS criteria = or > 2 (81.1% vs.28.8%, *p* < 0.001), temperature (38.1 ± 1.2 vs. 37.2 ± 1.9, *p* = 0.039), pulse rate(114.7 ± 20.7 vs. 100 ± 25.8, *p* < 0.001), respiratory rate (21.4 ± 10.9 vs. 18.2 ± 6.5, *p* = 0.018), WBC (16.2 ± 6.4 vs. 12.9 ± 4.7, *p* < 0.001), C-reactive protein (53.8% vs. 41.7%, *p* = 0.020), procalcitonin level (37.3% vs. 16.7%, *p* = 0.001), confirmed infection (79.1% vs. 6.0%, *p* < 0.001), requiring mechanical ventilation (96.4% vs. 78.5%, *p* < 0.001), and ICU days (6.1 ± 6.3 vs. 3.8 ± 6.6, *p* = 0.000) in the sepsis after sTBI group than in the non-sepsis after sTBI group. After adjusting the Cox regression analysis, only elevated CT score (hazard ratio [HR], 4.6; 95% CI, 3.373–10.49; *p* < 0.001) and elevated SOFA scores (HR, 3.0; 95% CI, 2.054–4.826; *p* < 0.001) was shown to be the good predictors of mortality for sepsis after sTBI (Table 3). The later positive bacterial culture in sputum or blood is showed in sTable 1 in the supplement.

**Table 1:** Baseline characteristics of 249 sepsis after sTBI on ICU

Characteristics	Total (*n* =249)
Male, n. (%)	163(65.5)
Age (years)	69(23–82)
Prior history, n. (%)	
Hypertension	114(45.8)
cardiac-cerebral vascular disease	91(36.7)
Chronic pulmonary disease	86(34.6)
Diabetes	40(16.2)
Cancer	22(8.8)
Findings of CT screening before on ICU, n, (%)	
Elevated brain CT score	236(94.8)
Elevated lung CT score	227(91.2)
Elevated abdominal CT score	50(20.1)
Both acute brain and lung edema	335(89.2)
Site of infection, n.(%)	
Community-acquired pneumonia/infection	197(79.1)
Abdominal infection	50(20.1)
CNS infection	2(0.8)
Median(IQR) time from onset to CT screening(hours)	1(0.5–229)
Median Time from onset to ICU(hours)	3.2(0.5–230)
LOS in ICU(day, medians[IQR])	9 (1–127)
Invasively mechanical ventilation	238(95.6)
Mortality in sepsis at 60 days follow-up	185(74.3)

*Abbreviation*: *ICU* intensive care unit, *GCS* Glasgow Coma Scale, *LOS* length of stay

**Table 2:** The results of univariate analysis in sepsis after sTBI patients and Non- sepsis after sTBI during the 30 days (*n*=412)

Variable	TBI with sepsis (249)	TBI without sepsis (*n* =163)	*P* Value
Male gender, n (%)	99(72.8)	97(76.4)	0.572
Age (years, mean ±SD)	56.1±16.4	52.7±16.6	0.095
Median time from injury to ICU (h, range)	1(2.5)	1(2.5)	0.39
GCS score (mean±SD)	5.0±1.8	6.0±1.9	0.027
Mean arterial pressure (mmHg, mean ±SD)	100.8±29.2	101.2±26.7	0.926
Respiratory rate (breaths/mim, mean±SD)	21.4±10.9	18.2±6.5	0.018
Body temperature (℃, mean±SD)	38.1± 1.2	37.2± 1.9	0.003
Heart rate (beats/min, mean±SD)	114.7±20.7	100±25.8	0
leukocyte count (x10^9^/l, mean±SD)	16.2±6.4	12.9±4.7	0
SIRS >2, n (%)	81.1	28.8	0
Blood glucose (mmol/l, mean ±SD)	9.7±3.4	9.1±3.6	0.244
Blood lactic acid (mmol/l, mean ±SD)	3.2±2.5	3.1±2.5	0.324
SOFA score (mean±SD)	5.9±2.2	3.6±0.5	0
C-reactive protein, n (%)	53.8	41.7	0.02
Procalcitonin, n (%)	37.3	16.7	0
CAP/infection, n (%)	79.1	6	0
Elevated CT score (mean±SD)	3.5± 1.4	0.9± 1.1	0
ICU days (mean±SD)	6.1±6.3	3.8±6.5	0
Mechanical ventilation, n (%)	96.4	78.5	0
At 60 days follow-up, n (%)			
GOSE=4–8	12.4	36.8	0
GOSE=1	74.3	44.8	0

*Abbreviation*: *ICU* intensive care unit, *TBI* traumatic brain injury, *SIRS* systemic inflammatory response syndrome, *SOFA* sequential organ failure assessment, *CAP* Community-acquired pneumonia, *GCS* Glasgow Coma Scale, *GOSE *Glasgow Outcome scale Extension

**Table 3:** Cox regression analysis in sepsis after sTBI patient with (*n*=412)

Variable	HR	95% CI for HR	*p * value
Elevated CT score	4.6	3.373–10.49	0
Elevated SOFA score	3	2.054–4.826	0

*Abbreviation*: *SOFA* sequential organ failure assessment

**Fig. 3:**
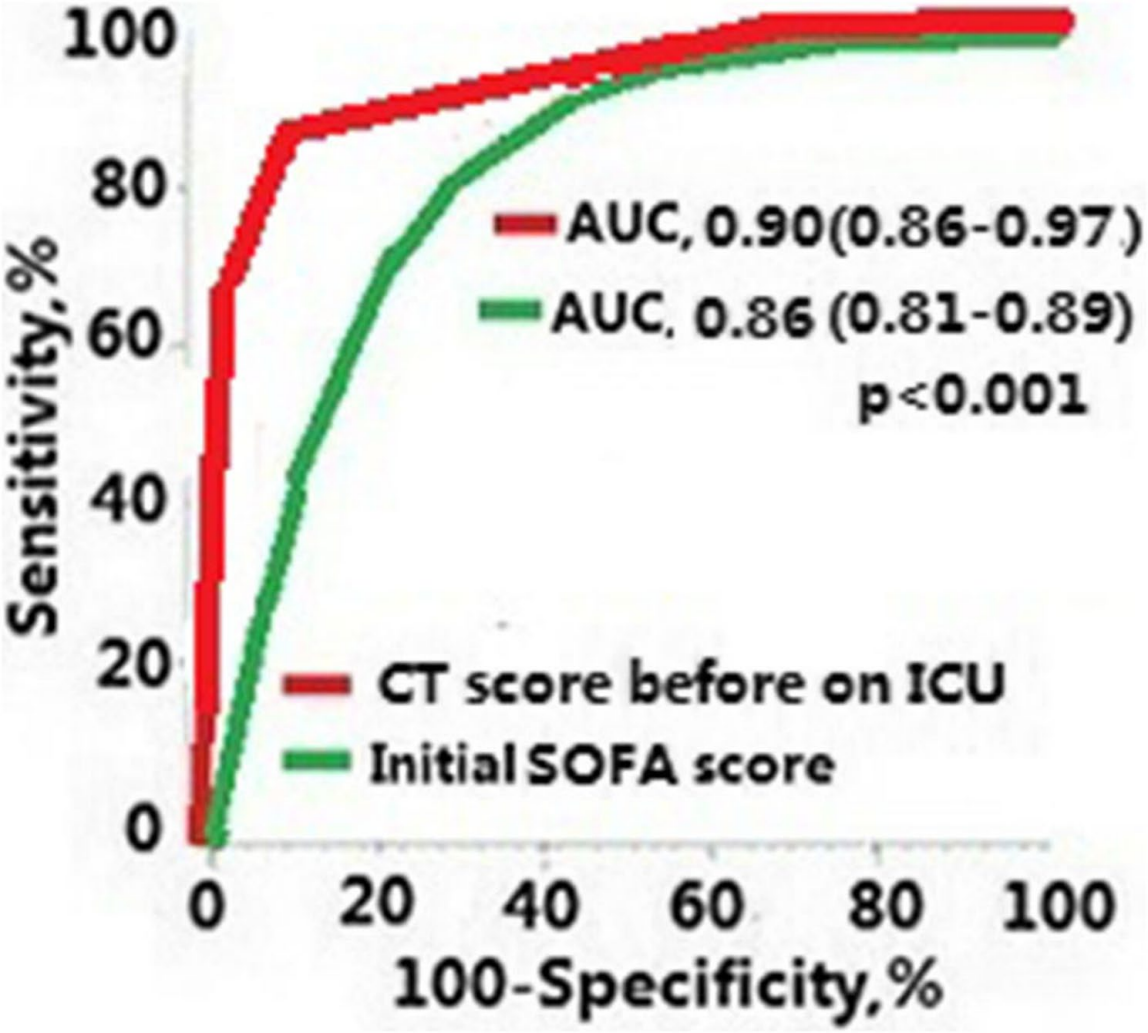
Comparisons of the areas under the receiver operating characteristic (ROC) curves for prediction of mortality

The elevated CT score presented the largest area under the ROC curve (0.90, 95%CI 0.86–0.97) compared with the elevated SOFA score (area under ROC curve 0.86, 95%CI 0.81–0.89). The area under the ROC curve was significantly larger for the CT score than for the SOFA score (*P* < 0.001, Fig. 3).

### Functional outcomes

**Fig. 4:**
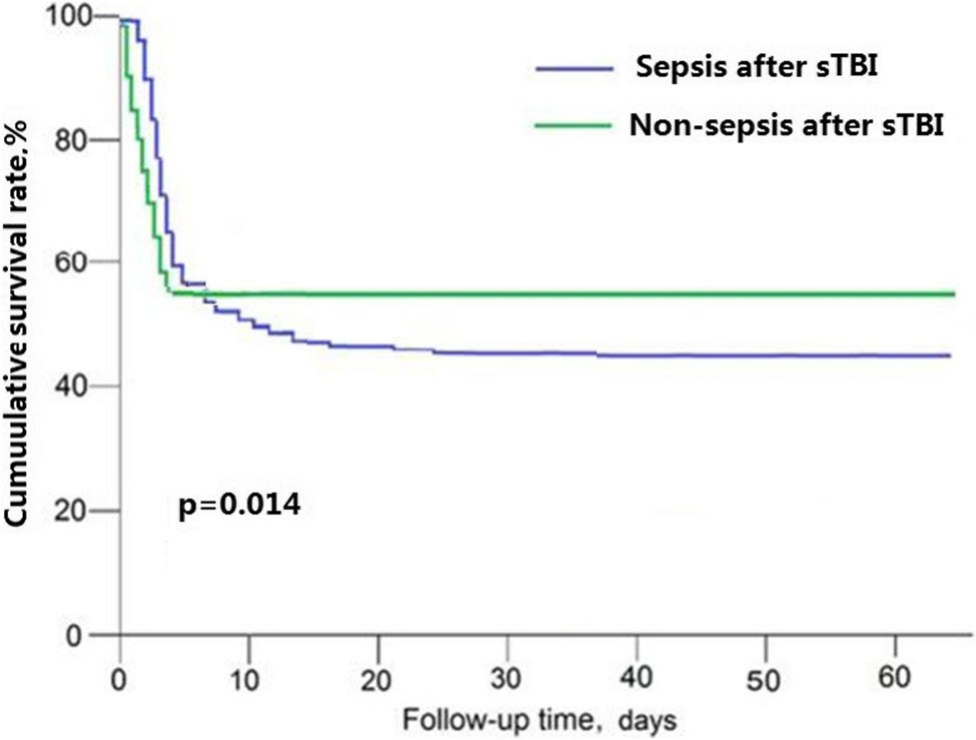
Kaplan-Meier survival curves for sepsis after sTBI and non- sepsis after sTBI. Kaplan–Meier survival curves show that sepsis after sTBI had significantly worse survival than those non-sepsis after sTBI during the 60days follow-up (*P* = 0.014)

There was a significant difference in the initial 60 day GOSE score between the two groups. The proportion of patients with good GOSE recovery in the sepsis after sTBI and the non-sepsis after sTBI was 12.4% and 36.8%, respectively. There was no significant difference in the proportion of patients in a vegetative state or with severe or moderate disabilities. However, there was a significant difference in the number of deaths between the two groups: the sepsis after sTBI was significantly higher than the non-sepsis after sTBI (74.3% vs. 44.8%, *p* < 0.001). The survival rates of the two groups of patients are shown in Table 3. During the initial 60 day follow-up, sepsis after sTBI patients had a significantly higher risk of death compared to non-sepsis after sTBI patients (*P* = 0.014) (Fig. 4). The elevated CT score in the area under the ROC curve for mortality was with 97.0% of sensitivity and 100.0% of specificity.

## Discussion

Previous studies have shown a high infection rate in TBI patients [13, 14]. In addition, the prevalence of sepsis in patients with traumatic brain injury increased from 10% in 1992 [3] to 75% in 2012 [15]. Currently, 60.4% of sepsis after sTBI patients are diagnosed according to the sepsis-3 ‘standard. Our current research indicates that before ICU admission, the incidence of sepsis after sTBI was also at the high end of the world. Previous TBI studies have mostly shown poor prognosis [16, 17], and the risk factors leading to death involve sepsis, which is related to MOF, mainly including respiratory and circulatory failure [2, 4]. In addition, most studies have evaluated patients with hospital/ICU acquired sepsis after sTBI, accompanied by MOF and poor outcomes [2, 16–18]. To our knowledge, pre-hospital sepsis after sTBI has not been well studied. However, before ICU admission, we used brain + chest + abdominal CT scans to quickly screen for sepsis after sTBI, which was not been reported by previous. We found that elevated CT score is closely related to the adverse outcome of sepsis after sTBI. These results are consistent with the hypothesis that elevated CT score leads to a poorer prognosis for sepsis after sTBI before admission to the ICU. We also found that CAP is the main cause of infection. Our current study indicates through adjusted Cox regression analysis that the elevated CT scare as well as elevated SOFA scores are the high-risk factor of death for sepsis after sTBI. The increase in SOFA scores mainly involves MOF, strongly indicating that this is a high-risk factor for life-threatening organ dysfunction in sepsis after sTBI. Although the sTBI event represents a life-threatening emergency, craniotomy decompression surgery for TBI patients has been reported [18–21]. TBI patients who received randomized treatment had showed poor prognosis [14, 15]. The mortality rate of sepsis was as high as 76% [16]. Our current study also showed similar mortality. However, in our study, Cox risk analysis confirmed that the life threatening risk of death from sepsis after sTBI was more than 4 times. The harsh reality indicates that the prognosis and mortality of sepsis after sTBI are mainly affected by sepsis accompanied acute brain injury and acute lung injury, rather than simple head injuries.

Several mechanisms may explain the low survival rate of sepsis after sTBI. (1) SIRS and immune response is very common in sepsis after sTBI patients, which may almost lead to the destruction of the broader Blood–brain barrier, thus exacerbating systemic ischemic injury/edema, and leading to life-threatening organ failure; [22–24] (2) Septic shock may have severe cerebral microcirculation disorders [25], leading to extensive subcortical white matter lesions or multifocal necrotic encephalopathy; [26] (3) The brain dysfunction associated with sepsis caused by CAP and severe hypoxia is also a fact. The coma from hypoxic brain injury is caused by acute brain edema or acute lung edema; and (4) MOF may jointly affect the host, leading to severe brain injury, and increasing the risk of death. The current research has found that most sTBI patients with sepsis before ICU admission by rapid CT screening shows that severe CAP is more likely to have acute brain injury due to ARDS, which is still understudied. However, a new evident that the high mortality of sepsis-associated brain injury related to ARDS due to infection of after SARS-CoV2 inCOVID-19 has been reported by Li et al. and Qin et al. [27, 28]. Thus, this also supputted that after sTBI due to SRAS caused high mortality of sepsis associated brain injury was not impossible.

Although our data comes from prospective registration, due to retrospective research, some limitations must be considered. First, in this cohort, one limitation is the single central design, which may present a selection bias. Second, the limitation of patients with sTBI may be an overestimation of the incidence rate of sepsis. Third, some patients are excluded because they died within 24 h in the ICU. Most of those patients could have sepsis after sTBI. Thus, this could overlook early mortality trends associated with sepsis with sTBI. Fourthly, Most sTBI patient were underwent an emergency craniotomy and external drainage. Indeed, we have not collected the date of examination in CSF. Thus, a diagnosis of secondary CNS infection may be missed In addition, sepsis related brain dysfunction is very common, with subcortical white matter ischaemic lesions and micro infarctions confirmed by MRI [29]. However, MR imaging scans were rarely performed in this study. Therefore, our CT data on the proportion of sepsis related brain injury may be underestimated.

In conclusion, elevated CT score and SOFA score in the ICU is a well indicator of high mortality and poor outcome for sepsis after sTBI, which suggests that this very current and practical event is involved to a global health care problem. But it could still need further verification in future investigation.

## Supplementary Information

Supplementary Material 1.

## Data Availability

No datasets were generated or analysed during the current study.

## Abbreviations

ICU: Intensive care unit
TBI: Traumatic brain injury
SIRS: Systemic inflammatory response syndrome
ABE: Acute brain edema
ALE: Acute lung edema
SOFA: Sequential organ failure assessment
GCS: Glasgow Coma Scale
GOSE: Glasgow Outcome Scale-Extended
