# Supplementary material for: Sepsis in patients with severe TBI: a retrospective CT scoring study
Source: Int J Emerg Med. 2025 Jun 23;18:111. doi: 10.1186/s12245-025-00911-6 (PMC12183873; doi:10.1186/s12245-025-00911-6)
Supplement: Supplementary file 1 — Supplementary Material 1. [file 12245_2025_911_MOESM1_ESM.doc]

# [**Supplementary Appendix**](https://www.nejm.org/doi/suppl/10.1056/NEJMoa1502826/suppl_file/nejmoa1502826_appendix.pdf)

**Supplementary to:** sepsis after severe TBI

**Additional: sTable 1. Condition of infection in sepsis after sTBI** (n=412)

|  | Total  (n=412) |
| --- | --- |
| Later culture and CSF analysis ,n(%) |  |
| §Positive CSF analysis  Positive urine culture  **Positive sputum culture, n(%)**  *Acinetobacter baumannii*  *Klebsiella pneumonae*  *E.coli*  *Staphylococcus aureus*  **Positive blood culture, n(%)**  Gram negative bacilli  *Acinetobacter baumannii*  *Klebsiella pneumonae*  *Pseudomonas aeuruginosa*  *E.coli*  *Heamephilus influenzae*  Gram positive cocci  *Staphylococcus aureus*  *Streplococcus pneumoniac*  *Enteroccus feecalis*  *Fungi* | 34(8.3)  18(4.)  346(84.0)  102(29.5)  90(26.0)  63(18.2)  35(10.1)  118(28.6)  56 (47.5)  18(15.3)  15(12.7)  11(9.3)  10(8.5)  2(1.9)  48(40.7)  19(16.0)  16(13.6)  14(11.9)  6(5.1) |

Abbreviations: ICU, intensive care unit; CNS, central nervous system;CSF, cerebrospinal fluid;

§ confirmed by CSF: elevated leukocyte counts (median 386**×**10^6^**/**L，range from 50 to 740**×**10^6^**/**L), elevated CSF protein level (median 2.8 g/L, range from 0.88 to 3.0 g/L).

**Additional file 2: sTable 2.** The analysis of Glasgow Outcome Scale-Extended for sepsis in patients with TBI at 60days follow-up

| Variable, n (%) | Sepsis after sTBI  (N=249) | Non-sepsis after sTBI  (N=163) | *P*  Value |
| --- | --- | --- | --- |
| 1.Dead  2.Vegetative state  3-4 Lower or upper severe disability  5-6.Lower or upper moderate disability  7-8 Lower or upper good recovery | 185(74.3)  19(7.6)  8(3.2)  6(2.4)  31(12.4) | 73(44.9)  12(7.4)  8(4.9)  8(4.9)  60(36.8) | ＜0.001  1.000  0.459  0.178  ＜0.001 |

Abbreviation: TBI, traumatic brain injury; ICU, intensive care unit; GOSE, Glasgow Outcome Scale-Extended.

sTable 3.Baseline characteristics of 412 patients with TBI in ICU

| Baseline characteristics | Value |
| --- | --- |
| Sex, n. (%)  Male  Female | 315(76.5)  97(23.5) |
| Age,yr (mean±SD)  Median time from injury to ICU (hours, range) | 53±16.4  1(3.0) |
| Injure Mechanism, n. (%)  Road traffic collisions  Falls  Cycling falls  Falls from same level  Falls from height  Falls from Epilepsy  Unknown  Median GCS score on admision,(range)  3-4 score,n. (%)  5-6 score, n. (%)  7-8 score, n. (%) | 247(59.9)  146(35.4)  51(35.2)  40(27.8)  39(24.0)  2(0.1)  20(4.6)  6(3-8)  193(46.8)  56(13.7)  163(39.5) |
| Head CT scan findings, n. (%)  Subarachnoid hemorrhage  Contusions  Subdural hematoma | 382(92.8))  277(67.3)  228(55.5) |
| Intracerebral hemorrhage  Epideural hematoma  Other  Mechanical ventilation, n (%)  Surgery, n. (%) | 210(51.0)  180(43.7)  89(21.7))  267(89.0) |
|  |  |
|  |  |
|  |  |

Abbreviations:TBI, traumatic brain injury; ICU, intensive care unit;
